# Office Space Bacterial Abundance and Diversity in Three Metropolitan Areas

**DOI:** 10.1371/journal.pone.0037849

**Published:** 2012-05-30

**Authors:** Krissi M. Hewitt, Charles P. Gerba, Sheri L. Maxwell, Scott T. Kelley

**Affiliations:** 1 Department of Biology, San Diego State University, San Diego, California, United States of America; 2 Department of Soil, Water and Environmental Science, University of Arizona, Tucson, Arizona, United States of America; Institute for Genome Sciences, University of Maryland School of Medicine, United States of America

## Abstract

People in developed countries spend approximately 90% of their lives indoors, yet we know little about the source and diversity of microbes in built environments. In this study, we combined culture-based cell counting and multiplexed pyrosequencing of environmental ribosomal RNA (rRNA) gene sequences to investigate office space bacterial diversity in three metropolitan areas. Five surfaces common to all offices were sampled using sterile double-tipped swabs, one tip for culturing and one for DNA extraction, in 30 different offices per city (90 offices, 450 total samples). 16S rRNA gene sequences were PCR amplified using bar-coded “universal” bacterial primers from 54 of the surfaces (18 per city) and pooled for pyrosequencing. A three-factorial Analysis of Variance (ANOVA) found significant differences in viable bacterial abundance between offices inhabited by men or women, among the various surface types, and among cities. Multiplex pyrosequencing identified more than 500 bacterial genera from 20 different bacterial divisions. The most abundant of these genera tended to be common inhabitants of human skin, nasal, oral or intestinal cavities. Other commonly occurring genera appeared to have environmental origins (e.g., soils). There were no significant differences in the bacterial diversity between offices inhabited by men or women or among surfaces, but the bacterial community diversity of the Tucson samples was clearly distinguishable from that of New York and San Francisco, which were indistinguishable. Overall, our comprehensive molecular analysis of office building microbial diversity shows the potential of these methods for studying patterns and origins of indoor bacterial contamination. **“**[H]umans move through a sea of microbial life that is seldom perceived except in the context of potential disease and decay.” – Feazel et al. (2009).

## Introduction

In the industrialized world, millions of people spend their entire working day, eight or more hours, inside office buildings sometimes without going outdoors the entire day [1]. Employees in crowded buildings often share workstations, computers, chairs, restrooms and many other common areas that have been found to harbor a wide spectrum of microorganisms. Studies of office building air have detected as many as 10^6^ bacteria per cubic meter [2], and the constant influx of microbes brought in with office workers likely makes for a dynamic microbial environment [3]. Human skin, as well as oral and nasal cavities, harbor trillions of microorganisms that may be shed and accumulate in offices [4]–[6]. Microbes from soils or other environments can also be vectored by office workers or be carried on dust particles from the outdoor air [7]. Moreover, indoor office buildings offer unique chemical environments not encountered in the natural world that may enrich for particular microbes [8].

While humans are increasingly spending more of their lives in office buildings, we remain relatively ignorant concerning the microbial diversity of these habitats [3]. Culture-based microbiology studies have shown that viable microorganisms are readily obtained from offices and other indoor environments, such as schools, houses, hospitals and restrooms [9]–[14]. Culture-based studies indicate that Gram-positive bacilli tend to dominate indoor environments, along with a few Gram-negative species including *Chryseomonas* spp. and *Pantoea* spp. [12], [13]. Indoor culture studies have also identified many Actinomycetes, such as *Rhodococcus fasclans*, *Arthrobacter pascens*, and *Corynebacterium* spp., as common inhabitants of built environments [12]. Although culture-based studies can verify the viability of at least some microbes in a given environment, it has long been known that culturing studies capture only a small proportion (<1%) of the existing microbiological diversity [15]–[19].

Culture-independent molecular studies based on small-subunit ribosomal RNA (16S rRNA) gene sequences have greatly expanded our understanding of the bacterial diversity in indoor settings, such as houses [20], indoor pools [21], airplanes [22], [23], and daycares [24]. These studies have revealed an enormous diversity of microbes, several orders of magnitude greater than detected via culturing. In some cases, culture-independent methods have identified many potential pathogens or opportunistic pathogens [2], [21], [25]. In 2008, a 16S rRNA based study of bacterial diversity in two different office buildings in Finland, discovered hundreds of unique microbial lineages (OTUs) from 8 clone libraries sampled in all four seasons of the year [3]. The authors found strong seasonal dynamics and large differences in the diversity of the two offices buildings. However, the study was limited by the time and expense of clone library construction and sequencing, and more work needs to be done to understand how these results generalize to other office settings.

In the past few years, researchers have successfully applied multiplexed high-throughput sequencing technologies to sequence thousands of 16S rRNA gene sequences from dozens or hundreds of environments simultaneously – up to 100 times more sequences per sample than typical clone library studies [26], [27]. These methods have been applied to study human disease [6] and natural microbial environments [26], [28]. (See [29] for an extensive list of studies). The combination of the culture-independent 16S rRNA-based methods and multiplexed pyrosequencing approaches has created a so-called “renaissance” for the 16S rRNA approach to investigating microbial diversity [29]. In this study, we used a combination of multiplex pyrosequencing of 16S rRNA gene sequences and heterotrophic viability cell-counting assays to gain a deeper understanding of the composition and abundance of bacterial contamination in modern office buildings. Specifically, we surveyed office building contamination in New York, San Francisco and Tucson, Arizona by swabbing five common surface types in thirty randomly chosen offices. These cities represented three diverse climatic regimes, allowing for a broader generalization of what constitutes “typical” office building microbial diversity. The five surfaces were chosen because they are commonly found in offices and also because they represent a diverse set frequently touched surfaces. We used culture-based methods to estimate heterotrophic bacterial abundance and amplified bacterial 16S rRNA gene sequences via PCR with “universal” bar-coded PCR primers from a subset of the samples. Our three-factorial sampling design allowed us to determine the effects of city, surface, and the gender of office occupants (hereafter referred to as simply “gender”) on the abundance of heterotrophic bacterial contamination. The high-throughput multiplexed pyrosequencing analysis allowed us to establish a highly detailed picture of office building contamination and determine how city, surface and gender correlated with bacterial surface contamination.

## Methods

### Sample Collection

Samples were collected from the same five surfaces in 90 randomly chosen offices in three different office buildings located in New York, NY; San Francisco, CA; and Tucson, AZ, half inhabited by men and half by women. In each office we swabbed approximately 13 cm^2^ of the same five surface types: chairs, phones, computer mice, computer keyboards, and desktops. Environmental samples were taken with dual tip sterile cotton swabs (BBL CultureSwab™, catalog # 220135, Becton Dickinson, Sparks, MD) and these were stored in sterile-labeled tubes, placed on ice and shipped overnight to the lab at the University of Arizona. One of the dual tip swabs was used to count viable heterotrophic bacteria while the remaining swab was used for DNA extraction and PCR analysis. Sampling did not directly involve human subjects (e.g., sampling of human skin, nostrils), only the collection of dust and biofilm on inert surfaces. However, we did note the gender of the occupant in each office. The sampled buildings were not restricted spaces and no special permits were required to obtain samples.

### Cell Count Analysis

The numbers of heterotrophic bacteria (HPC) were determined on R2A media (Difco, Sparks, MD) using the spread plate method. Samples were diluted using physiological saline for assay of 10^−1^ through 10^−3^ dilutions. All dilutions were assayed in duplicate. The plates were then incubated at 30°C for 5 days and colonies counted.

### DNA Extraction and PCR

Prior to DNA extraction, the cotton from the swab was removed using a flame-sterilized razor blade and the cotton threads were placed into a lysozyme reaction mixture [30]. The reaction mixture had a total volume of 200 µl and included the following final concentration: 20 mM Tris, 2 mM EDTA (pH 8.0), 1.2% NP-40 detergent, 20 mg ml^−1^ lysozyme, and 0.2 µm filtered sterile water (Sigma Chemical Co., St. Louis, MO). Samples were incubated in a 37°C water bath for thirty minutes. Next, Proteinase K (DNeasy Tissue Kit, Qiagen Corporation, Valencia, CA) and AL Buffer (DNeasy Tissue Kit, Qiagen Corporation, Valencia, CA) were added to the tubes and gently mixed. Samples were incubated in a 70°C water bath for 10 min. All samples were purified spin columns from a DNeasy Tissue Kit (Qiagen; following [24]). After purification, the DNA was quantified using a NanoDrop ND-1000 Spectrophotometer (NanoDrop Technologies, Willmington, DE).

PCR amplifications were performed on the 54 chair and phone surface samples (18 per city, 9 from men’s offices and 9 from women’s), which, on average, were the most contaminated according to the cell counting assay. The “universal” bacterial PCR primers had been previously designed from regions of the 16S rRNA gene conserved in all bacteria (27F and 338R) and the same primer set has been used in numerous other studies [29], [31]. The primers flank a highly variable region of the 16S rRNA gene sequence that is ideal for pyrosequencing studies [31]. The 338R primers were also designed with a 12-nucleotide “barcode” unique to each sample. The sequence barcode allowed all the PCR products to be pooled into one 454 sequencing run. The forward primer included a short sequence necessary for the pyrosequencing reaction. PCR reactions were carried out in a total volume of 50 µl including 1 µl (approx. 10 ng µl^−1^) of sample DNA as template, 400 µM of each deoxynucleoside triphosphate, 1.65 mM MgCl_2,_ 5 µl 10× buffer (10× concentration: 500 mM 1 M KCl, 100 mM 1 M Tris HCl pH 8.4, 1% Triton-X), 1 µM of each primer, and 1 µl of REDTAQ™ DNA polymerase (1 unit µl^−1^; Sigma-Aldrich Inc., St. Louis, MO). Thirty cycles of PCR amplification were performed for the environmental swab samples. All PCR cycles included an initial denaturation step at 94°C for 1 min, an annealing step at 55°C for 45 sec and an extension step at 72°C for 1.5 min. The amplification cycles were preceded by a one-time denaturing step at 94°C for 5 min prior to the first cycle and included a final 72°C extension for 10 min to ensure complete extension.

### Sequencing

Individual barcoded PCR products were purified using the AMPure purification kit (Agenourt, Beverly, MA) following the manufacturer’s protocol. After AMPure purification each sample was quantified on an Agilent 2100 Bioanalyzer. All samples were diluted down to 2×10^−5^ moles/µL^−1^ (50 µL volume) and were then pooled with a total combined concentration of 2×10^−5^ moles/µL^−1^ (100 µL total volume). PCR purification, dilutions and pyrosequencing on a 454 Life Sciences FLX Genome Sequencer were all conducted by the core facility at the University of South Carolina (Environmental Genomics Core Facility).

### Computational and Statistical Analyses

Bacterial count data were analyzed using Systat (version 12; Systat Software, Inc. Chicago, IL). Because the data were not normally distributed, the counts were ranked across the entire dataset and then analyzed using a 3-way (non-parametric) ANOVA.

Analysis of the multiplexed bar-coded pyrosequencing data was performed using the Mac implementation of the QIIME package [32], an integrated platform for analysis of microbial 16S rRNA gene sequences, with which we performed the following quality controls and analyses using the default parameters. Sequences were split into samples by barcodes, and low quality reads were filtered, leaving only high-quality sequences (>200 bp in length, quality scores >25 and exact barcode and primer matches), which were then denoised. Each library was rarified down to the same sequencing depth (1000 sequences) to mitigate sample depth bias, and clustered into OTUs (97% sequencing identity) using UCLUST [33]. Representative sequences for each cluster were aligned against the Greengenes core dataset [34] using PyNAST [35] and taxonomy was assigned via the RDP-classifier [36]. The FastTree algorithm was used to make the phylogenetic trees [37], which were subsequently used for beta-diversity (weighted UniFrac) [38] and Principal Coordinate Analyses (PCoA).

## Results

**Figure 1:**
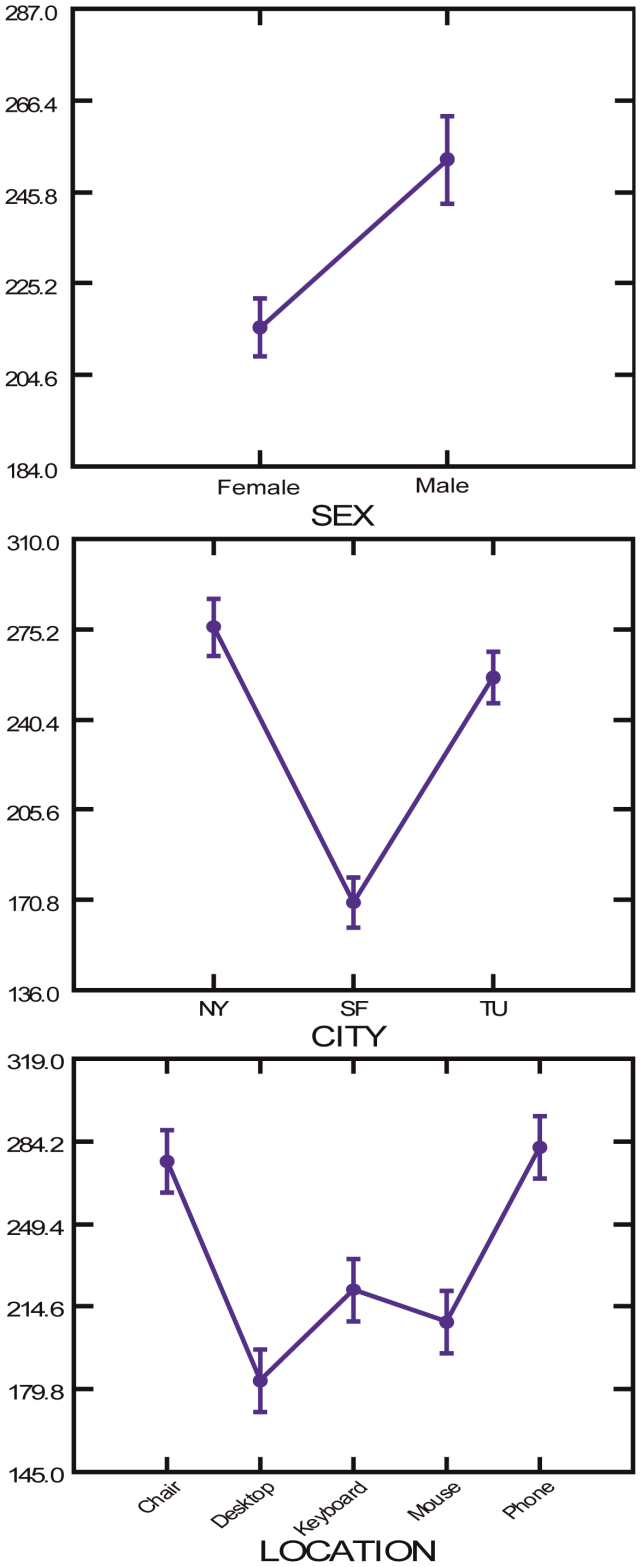
Transition graph showing the average bacterial counts between genders, among cities and among office locations. The dots indicate the mean bacterial abundance for surfaces grouped by gender of office occupant (top graph), by city (middle graph) and by surface type (bottom graph). The lines connect the means and standard errors for the ranked bacterial counts (see Methods).

Viable heterotrophic bacteria were cultivated off nearly every surface. One-way ANOVAs found highly significant differences in bacterial abundance among cities (Table 1: *F*_2,495_ = 31.71; P<0.001), between the offices inhabited by men and women (*F*_1,495_ = 10.295; P = 0.001) and among office surfaces (*F*_4,495_ = 10.661; P<0.001). Of the possible 2-way and 3-way interactions, there was only one significant 2-way interaction, that between City and Surfaces (Table 1: *F*_8,495_ = 2.574; P = 0.009). Figure 1 shows the means and standard errors for each of the various sample groups for the ranked bacterial counts. The values were transformed to rank order values (non-parametric) because the counts were not normally distributed. The transition graphs illustrate clear differences between samples, and the general lack of higher order interactions makes these data readily interpretable.

**Table 1:** Results of three-way ANOVA examining the effects of city, gender of office inhabitant, and surface sample location on bacterial cell abundance.

Source	Sums-Sq	df	Mean-Sq	F	P
**Main Effects**					
City^1^	829726.016	2	414863.008	31.71	<0.001
Gender^2^	134685.392	1	134685.392	10.295	0.001
Location^3^	557911.089	4	139477.772	10.661	<0.001
**2-way Interactions**					
City * Gender	33140.228	2	16570.114	1.267	0.283
City * Location	269388.829	8	33673.604	2.574	0.009
Gender * Location	29958.987	4	7489.747	0.572	0.683
**3-way Interations**					
City * Gender * Location	82164.06	8	10270.508	0.785	0.616

1 New York, San Francisco, Tucson;

2 Male, Female;

3 Chair, Desktop, Keyboard, Mouse, Phone.

The DNA extractions for all 54 swabs contained measurable quantities of bacterial DNA (4–10 ng µl^−1^), except the negative extraction controls, which had no quantifiable DNA. Subsequent PCR reactions were performed in small lots (six reactions plus positive and negative DNA extraction controls) to reduce the possibility of contamination. All samples produced visible PCR products except the negative PCR and DNA extraction controls.

**Figure 2:**
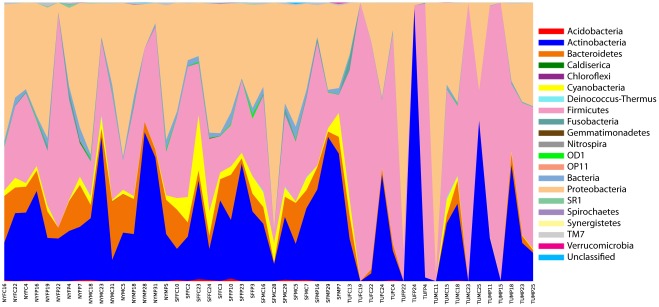
Relative abundance of bacterial divisions across samples. The abundances of various bacterial divisions (see color legend) in the 54 samples were based on multiplexed pyrosequencing of 16S rRNA gene sequences. The codes for each sample are presented along the X-axis and indicate the city (NY = New York, SF = San Francisco, TU = Tucson), gender of the office occupant (F = Female, M = Male), and site within the office from which the sample (C = Chair, P = Phone) was obtained, followed by sample number.

**Figure 3:**
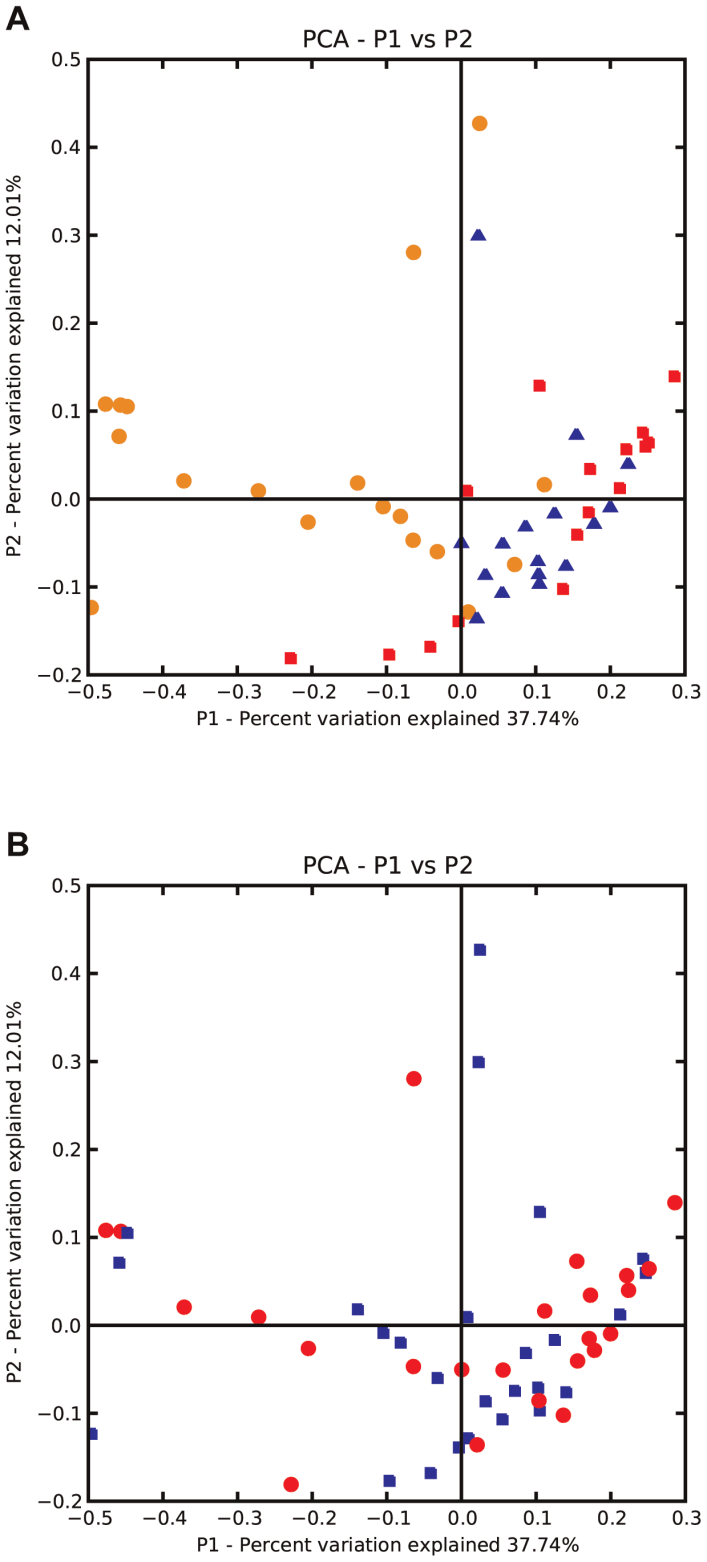
Principal Coordinates Analysis (PCoA) of the weighted pair-wise Unifrac distances between samples. The first two principal coordinates explain approx. 50% of the variation. (A) Samples coded by city: Blue Triangles = New York; Red Squares = San Francisco; Orange Circles = Tucson. (B) Samples coded by gender of office occupant: Red Circle = Female; Blue Square = Male.

A pyrosequencing reaction (half-run) of the 54 surface swab samples yielded a total of 177,000 sequences with an average of 239 bp (43.5 Mb of data). There were approximately 140,918 sequences >200 bp in length (median length 250 bp) left after quality and chimera checking and removal of the low quality reads. After adjusting the sampling depth to 1000, we determined 3865 distinct OTUs at the 97% similarity level belonging to different bacterial genera across 20 bacterial divisions. Figure 2 displays the relative abundances of various bacterial divisions in each of the samples. Proteobacteria, Firmicutes and Actinobacteria were consistently the most prominent across all samples. Figure S1 presents a breakdown of the specific bacterial taxonomic groups (minimum 50 OTUs) found across all samples. Figure 3 show a Principal Coordinates Analysis (PCoA) of the weighted pair-wise Unifrac distances between the various samples. The first two principal coordinates together explained ∼50% of the variation in weighted pair-wise Unifrac distances between all samples. The first principal component explained almost 38% of the variation and appeared to correlate very strongly with the city of origin (Fig. 3A), while gender and surface type did not appear to correlate with any of the first three principal components (e.g., Fig. 3B; data not shown).

## Discussion

Our intensive sampling effort, combined with a robust 3-factorial statistical design, allowed us to make several strong conclusions concerning the factors most associated with heterotrophic bacterial abundance. We found highly significant differences in bacterial abundance (P≤0.001) among cities, surfaces and between genders (Table 1). Surfaces in offices inhabited by men were consistently more contaminated than those of offices inhabited by women (Table 1; Fig. 1). We also found that chairs and phones were the most contaminated of the five surfaces (although all surfaces were contaminated) and offices in San Francisco tended to be less contaminated than those in New York or Tucson (Fig. 1).

While the differences among cities do not seem readily interpretable, the differences between contamination levels in the offices of men and women may explained by differences in hygiene. Men are known to wash their hands and brush their teeth less frequently than women, and are commonly perceived to have a more slovenly nature [39], [40]. Given the high proportion of human-associated bacteria found on the surfaces by the culture-independent analysis (Fig. S1), the differences may also be partially attributable to body-size. Since men are, on average, larger than women, they have a correspondingly greater skin surface area, as well as nasal and oral cavities and, therefore, a proportionally greater surface area for bacterial colonization. Thus, in addition to being less hygienic, it is possible that men may also shed more bacteria into their surrounding environment.

As expected, the high-throughput multiplex pyrosequencing of bacterial 16S rRNA gene sequences allowed a rapid and thorough assessment of the microbial diversity. Altogether, we determined 16S rRNA gene sequences that matched more than 500 bacterial genera from 20 different bacterial divisions (Fig. S1; data not shown). Members of the Proteobacteria were most common on all surfaces, followed by Firmicutes, Actinobacteria and Bacteroidetes; taken together, these groups accounted for almost 90% of the sequences (Fig. 2; Fig. S1). Our ability to detect sequence diversity, although dramatically increased by the multiplex high-throughput methods, was still limited by the relatively short 454 sequencing reads and the evolutionary conservative nature of the 16S gene. We were confident of our determinations at the level of bacterial genera, but more precise detection of species or strains will require longer sequencing read-lengths or information from faster evolving genes. Our ability to detect representatives from so many bacterial divisions suggests that our PCR primers were reasonably universal for Bacteria, though we cannot altogether rule out PCR-bias especially as it might have impacted total abundance. However, the fact that we were able to detect so many spore-forming Firmicutes (e.g., Bacilli) and acid-fast Actinobacteria indicates that our DNA extraction procedures were effective with a wide diversity of cell types. Moreover, in terms of relative taxonomic abundance, our findings largely corresponded to other culture-independent studies of indoor environments that employed PCR cloning methods and other 16S rRNA primer pairs [3], [20], [24].

Humans were clearly the primary source of office bacterial contamination. Many of the most common genera we discovered inhabit human skin, oral or nasal cavities. For example, a previous high-throughput 16S rRNA study of human skin discovered many or our most prevalent bacterial genera, such as *Streptococcus*, *Corynebacterium*, *Flavimonas*, *Lactobacillus*, and members of the *Burkholderiales* [40]. A number of genera we determined in offices are commonly found in oral samples, such as *Prevotella*, *Neisseria*, *Pseudomonas*, *Actinomyces* and TM7 bacteria [41]. We also found a surprising number of bacterial genera associated with the human digestive tract, including members of the Bacteroidetes, as well as *Lactobacillus* and members of the Enterobacteriaceae [42], [43]. Although several of these genera include pathogens (e.g., *Neisseria*, *Shigella*, *Streptococcus*, *Staphylococcus*) and opportunistic pathogens, the sequence information we collected could not distinguish bacterial strains or species. However, most of the human-associated bacteria we found were likely commensals and would only be a potential problem with severely immune compromised individuals.

The other main source of bacteria contamination on office surfaces appeared to have been environmental in origin. Many genera we found are associated with soils (e.g., *Bacillus*), and the rhizosphere (e.g., *Bradyrhizobium*). Many of the common sequences we determined also matched poorly known genera from environmental sources, including *Planomicrobium*, *Planococcus* and Microbacteriaceae (Figure S1).

The types of microbes found were similar to those discovered in a study of seasonal office diversity in Finland [3]. We also found that Firmicutes tended to be the most abundant organisms on all surfaces, and that members of the Proteobacteria were extremely common. Overall, our study determined sequences from at least twice as many bacterial groups as the Finnish study (549 bacterial genera vs. 283 unique OTUs in the Finnish study). The differences in our results can likely be attributed to a combination of broader sampling and much deeper sequencing of PCR amplified 16S sequences. Our results were also similar to studies of airplane bacterial contamination, particularly in terms of the human-associated microbiota [22], [23]. These airplane contamination studies tended to find human-associated bacteria but found less soil-associated bacterial diversity. This make sense, given that airplanes are not exposed to the outside, except for very short periods of time, and may not tend to accumulate as many dirt and soil particles. In contrast, our results were dissimilar to the findings of several other 16S-based indoor environment studies, including studies of a child daycare facility [24], a hospital therapy pool [21], shower curtains [25] and showerheads [2]. Unlike offices, these habitats tended to be highly “enriched” in particular bacteria, such as *Pseudomonas* (daycare; [24]), *Mycobacterium* (pools [21] and showerheads [2]) and *Sphingomonas* or *Methylobacterium* (shower curtains [25]). Temperature and moisture conditions likely enriched for certain microbes in these environments, particularly in the therapy pools and showers. In contrast, indoor office surfaces tend to be extremely dry and cool making for poor growth conditions. These differences may also explain why we did not observe an overabundance of any particular bacterial type.

No clear associations appeared to exist between the bacterial diversity, per se, and either gender of the office occupant or surface types. The contamination in the offices of men and women generally had the same types of common bacteria in similar proportions (data not shown), and a PCoA analysis of the weighted pair-wise Unifrac distances did not detect any meaningful clustering of samples by gender or by surface types (Figure 3B; data not shown). On the other hand, the PCoA uncovered a strong separation of Tucson samples from the New York and San Francisco samples, correlated with the first principal component (Figure 3A). This difference is also clearly reflected in the relative diversity various bacterial divisions. Unlike the samples from the two other cities, Bacteroidetes and Cyanobacteria were virtually absent in Tucson samples, many of which were completely dominated by members of a single bacterial division (Figure 2).

The PCoA plot (Figure 3B), the division abundances (Figure 2), and the taxonomic distribution of sequences (Figure S1) collectively indicate that Tucson samples tended to be much more variable than the samples from the other cities. (PCoA plots of the unweighted UniFrac distances were also performed and yielded similar results.) A closer look at the bacterial diversity of the Tucson samples suggests that the differences may be attributable to climate. Tucson samples were particularly abundant with members of the *Paenibacillus*, *Planococcus* and other Firmicute soil bacteria. The high proportion of Firmicutes in particular may, thus, be a product of the desert soils in and around Tucson.

Interestingly, our deep-sequencing approach also uncovered rare instances of microbes more commonly found in hot spring environments. For instance, we found many of our samples contained sequences related to bacterial divisions containing many known thermophiles, such as Chloroflexi, Deinococcus-Thermus, OP11 and OD1. While these may seem rather odd groups to find in office buildings, we note that independent studies of other indoor settings (e.g., restrooms; [14]) also uncovered small numbers of sequences belonging to these same phylogenetic groups. This may simply be a reflection of the dispersal and survival ability of these hardy organisms, which are found in hot springs world-wide, including on isolated volcanic islands [44]. Our results suggest that deep sequencing studies of indoor settings provide a potential means of studying how readily particular microbes are able to disperse around the globe.

Overall, the deep-sequencing approach used in this study provided novel insight into the diversity of office building environments. The baseline information we gathered in this study on microbial diversity in nominally “healthy” buildings could prove useful down the road for identifying causes of various building sickness syndromes. For instance, the microbial diversity of samples collected in “sick” buildings could be analyzed for meaningful departures from otherwise healthy buildings, possibly identifying the source of building-related health problems. Naturally, a much more comprehensive culture-independent molecular analysis of buildings in many environments needs to be undertaken, similar in scope to the EPA’s BASE study [45], to be truly effective in this regard. However, this study represents a reasonable first step and a model design for future sampling. As these techniques become easier and less expensive, they will allow much broader geographical and temporal surveys of diversity in office building and other settings and recently developed metadata standards for the built environment will further allow deeper investigation of how various abiotic factors (e.g., humidity, HVAC system) impact office building microbial diversity. Longer sequencing read lengths, new genetic markers and other Metagenomic methods should also increase resolution at the species and strain levels.

## Supporting Information

Figure S1 **Taxonomic OTU abundance table produced by QIIME (Heat map) using UCLUST to identify 97% similar sequences and RDP to identify nearest taxonomic groups and the deepest level possible given the data.** A particular OTU had to appear a minimum of 50 times sum total in all samples to appear in the table. The number of genera increased to ∼500 when the minimum was reduced to 5 OTUs.
(TIF)
