# Supplementary figures and images for: Office Space Bacterial Abundance and Diversity in Three Metropolitan Areas
Source: PLoS One. 2012 May 30;7(5):e37849. doi: 10.1371/journal.pone.0037849 (PMC3364274; doi:10.1371/journal.pone.0037849)

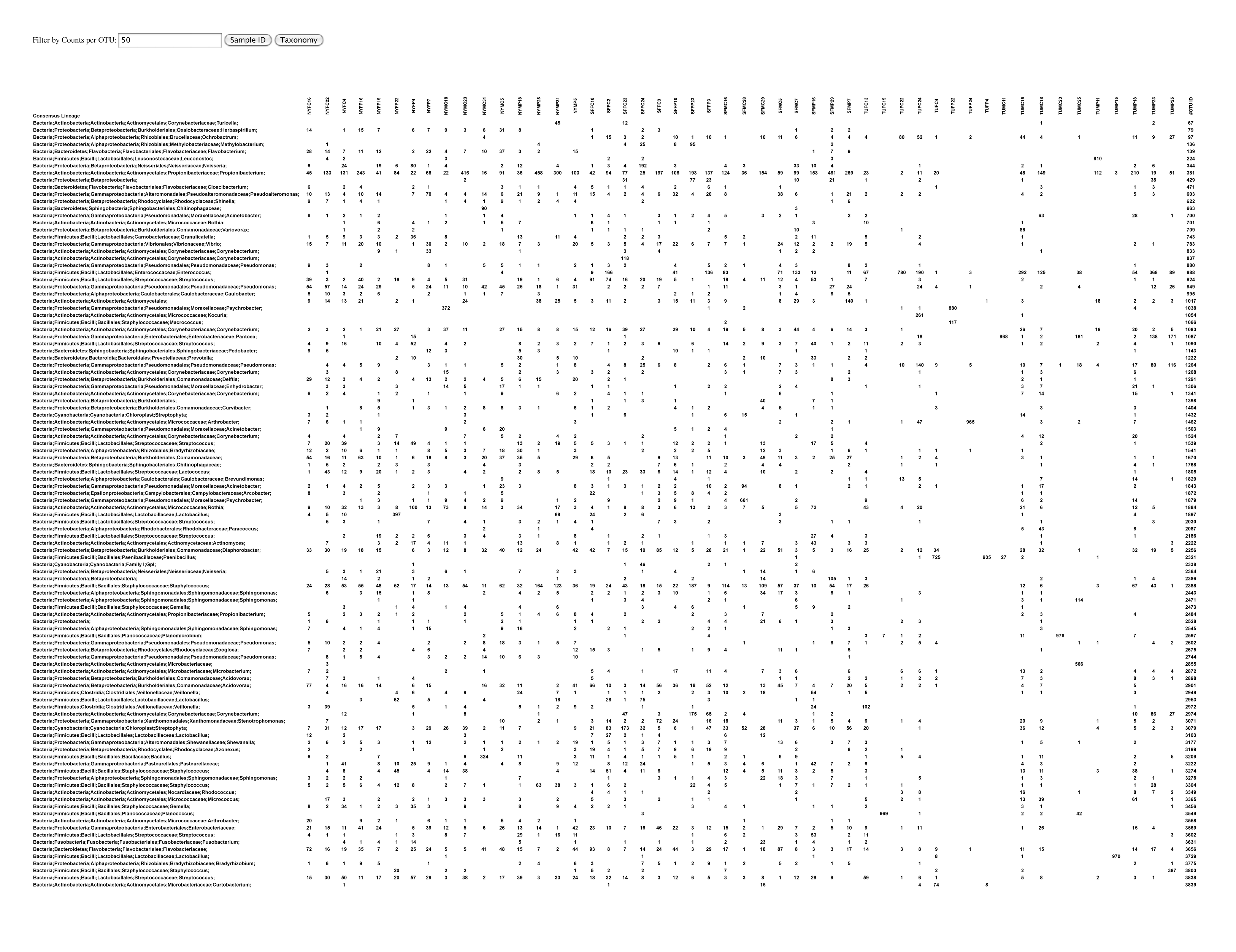

Supplement: Figure S1 — Taxonomic OTU abundance table produced by QIIME (Heat map) using UCLUST to identify 97% similar sequences and RDP to identify nearest taxonomic groups and the deepest level possible given the data. A particular OTU had to appear a minimum of 50 times sum total in all samples to appear in the table. The number of genera increased to ∼500 when the minimum was reduced to 5 OTUs. (TIF) [file pone.0037849.s001.tif]
